# dbDEMC: a database of differentially expressed miRNAs in human cancers

**DOI:** 10.1186/1471-2164-11-S4-S5

**Published:** 2010-12-02

**Authors:** Zhen Yang, Fei Ren, Changning Liu, Shunmin He, Gang Sun, Qian Gao, Lei Yao, Yangde Zhang, Ruoyu Miao, Ying Cao, Yi Zhao, Yang Zhong, Haitao Zhao

**Affiliations:** 1 School of Life Science, Fudan University, Shanghai, 200433, China; 2 Bioinformatics Research Group, Center for Advanced Computing Technology Research, Institute of Computing Technology, Chinese Academy of Sciences, Beijing, 100080, China; 3 Department of Liver Surgery, Peking Union Medical College Hospital, Chinese Academy of Medical Sciences, CAMS & PUMC, Beijing, 100730, China; 4 Central South University, Changsha, 410083, China; 5 Key Laboratory of the Zoological Systematics and Evolution, Institute of Zoology, Chinese Academy of Sciences, Beijing, 100101, China; 6 Key Laboratory of Medical Molecular Virology, Institutes of Biomedical Sciences and Institute of Medical Microbiology, Fudan University, Shanghai, 200032, China; 7 The Institute of Statistical Mathematics, 10-3 Midori-cho, Tachikawa, Tokyo, 190-8562, Japan; 8 Department of Biosystems Science, The Graduate University for Advanced Studies, Hayama, Kanagawa 240-0193, Japan; 9 Shanghai Center for Bioinformation Technology, Shanghai, 200235, China

## Abstract

**Background:**

MicroRNAs (miRNAs) are small noncoding RNAs about 22 nt long that negatively regulate gene expression at the post-transcriptional level. Their key effects on various biological processes, e.g., embryonic development, cell division, differentiation and apoptosis, are widely recognized. Evidence suggests that aberrant expression of miRNAs may contribute to many types of human diseases, including cancer. Here we present a database of differentially expressed miRNAs in human cancers (dbDEMC), to explore aberrantly expressed miRNAs among different cancers.

**Results:**

We collected the miRNA expression profiles of 14 cancer types, curated from 48 microarray data sets in peer-reviewed publications. The Significance Analysis of Microarrays method was used to retrieve the miRNAs that have dramatically different expression levels in cancers when compared to normal tissues. This database provides statistical results for differentially expressed miRNAs in each data set. A total of 607 differentially expressed miRNAs (590 mature miRNAs and 17 precursor miRNAs) were obtained in the current version of dbDEMC. Furthermore, low-throughput data from the same literature were also included in the database for validation. An easy-to-use web interface was designed for users. Annotations about each miRNA can be queried through miRNA ID or miRBase accession numbers, or can be browsed by different cancer types.

**Conclusions:**

This database is expected to be a valuable source for identification of cancer-related miRNAs, thereby helping with the improvement of classification, diagnosis and treatment of human cancers. All the information is freely available through http://159.226.118.44/dbDEMC/index.html.

## Background

Cancer, the leading cause of death worldwide, accounts for millions of deaths and huge economic burdens each year. One promising strategy for the diagnosis and treatment of cancer is to detect cancer-related biomarkers, which may have mutated or altered expression when comparing cancerous and normal tissues. Recently, a large amount of publicly available data at the genomic, transcriptomic and proteomic levels on cancers and corresponding exploration methods have greatly facilitated the identification of cancer-related biomarkers. Many cancer-related databases have been established to provide this kind of information, such as COSMIC [1], ITTACA [2], HPtaa [3] and dbDEPC [4]; however, most of them mainly focus on the protein-coding genes. In the recent decade, noncoding RNAs, especially the microRNAs (miRNAs), have been in the limelight of biomedical research. There is increasing evidence suggesting that the deregulation of miRNAs is associated with the development of many types of cancer [5-8], which made miRNAs a novel candidate biomarker for the diagnosis and treatment of human cancers.

miRNAs are small noncoding RNAs of about 22 nucleotides long which contribute to the post-transcriptional regulation of gene expression. They can inhibit the translation or strengthen the degradation of target transcripts through specific binding to messenger RNAs [9]. In the past few years, thousands of miRNAs have been identified in many organisms varying from viruses to mammals [10,11]. Their regulatory roles involved in different biological processes, such as cell proliferation, division, differentiation, apoptosis and embryo development, are extensively recognized [12-14]. The correlations between the alternation of miRNAs and the occurrence of human disease, especially in cancer, have been widely reported. The first evidence for the involvement of miRNAs in cancer formation was reported in 2002 [15]. Calin *et al.* found that miR-15 and miR-16 are located in a 30-kb region of chromosome 13q14, which is deleted in over 50% cases of B-cell chronic lymphocytic leukemia (B-CLL). The subsequent expression analysis indicated that both miRNAs were deleted or down-regulated in more than 68% of the cases. In another example, He *et al.* demonstrated that the enhanced expression of miR-17-92 cluster in B-cell lymphomas may accelerate c-Myc-induced tumorigenesis. This was the first time that the direct evidence for the involvement of miRNA in cancer was presented, thus miR-17-92 was referred to as oncomir-1 [16]. Interestingly, another work at the same time demonstrated that c-Myc could induce the expression of miR-17-92 and E2F1 growth factor, and that the miR-17-92 could inhibit the overexpression of E2F1; therefore, the miR-17-92 cluster could act as either an oncogene or tumor suppressor, depending on the context and cellular environment [17]. This miRNA cluster was also found to be up-regulated in lung cancer, while in contrast, another miRNA family, the let-7, was down-regulated [18,19]. In addition, downregulation of miR-122 was observed in hepatocellular carcinomas, whereas many other miRNAs were up-regulated [20]. It is speculated that more than 50% of the miRNA genes are located at cancer-related genome locus [21]. Many of them can act as oncogenes or tumor suppressor genes. Therefore, the identification of cancer-related miRNAs is of great importance for the diagnoses and treatment of human cancers.

High-throughput methods have been used to investigate miRNA expression pattern [22-24]. The expression profiles of miRNAs were believed to be more informative and accurate for classifying different cancer types in some cases [25]. As more and more transcriptomic data on miRNAs is published, several integrated resources for investigating and analyzing the expression patterns of miRNAs in normal tissues have been established, such as microRNA.org [26] and miRGator [27]. However, the number of databases concerning cancer-related miRNAs is still limited. To integrate the expression information of cancer-related miRNAs and improve the detection and classification of human cancers, we developed a publicly available database of differentially expressed miRNAs in human cancers (dbDEMC). This database integrated the expression data from 48 miRNA microarray data sets in peer-reviewed publications to provide the information of expression level change of miRNAs. In contrast to other databases, for example, the miR2Disease, which integrated miRNA-disease relationship mainly from the literature [28], results of dbDEMC were primarily retrieved from the analysis of high-throughput expression data, so that more quantitative information could be provided. Furthermore, dbDEMC is different from S-MED, which mainly focuses on the sarcoma-related miRNAs [29], in that dbDEMC provides miRNA expression for a broader spectrum of cancer cell lines. The establishment of this database will provide novel insight for cancer-related research. It could help with the identification of cancer-related miRNAs and further analysis of the roles that the miRNA plays in cancer formation, thereby helping with the improvement of classification, diagnosis and treatment of human cancers.

## Methods and results

### Data source

Differentially expressed miRNAs were curated from high-throughput data by a semiautomatic method. First, we searched the Gene Expression Omnibus (GEO) database using miRNA-related keywords, i.e., "miRNA" and "microRNA" and cancer-related keywords including "cancer", "tumor", "carcinoma" , "lymphoma" etc [30]. The search results were limited to those published before March 2010. For data quality control, only experiments with at least three biological replicates performed in both cancerous and normal tissues for single channel intensity data or at least three duplicates for two channel ratio data were selected. A total of 48 data sets from different cancer subtypes or cell lines of 14 cancers were chosen after the initial screen. Then the Significance Analysis of Microarrays (SAM) method was used to select miRNAs whose mean expression level is significantly different between cancer and normal tissues [31]. This method identified statistically differentially expressed genes by carrying out gene specific t-test. A "relative difference" score was computed for each gene. The D value was defined as the average expression change from different expression states to the standard deviation of measurements for that gene. Random permutation of the measurement was performed to estimate the false discovery rate (FDR). The siggenens package [http://www.bioconductor.Org/packages/2.3/bioc/html/siggenes.html] embedded in R was used to perform the SAM analysis. The miRNAs with Q value less than 0.05 were extracted as candidate genes that have significant different expression levels. In addition, some low-throughput methods, including the quantitative real-time PCR and northern blot, were used to validate the results of the microarray experiment; therefore, such information from the same literature was also incorporated into the database. miRNA identifiers were unified as miRBase IDs [32]. Each entry of the database contains the miRNA ID, miRBase accession number, miRNA sequence and detailed information about the analysis results. Considering the complexity of expression status of a specific miRNA from different experiments or from different subtypes or cell lines derived from the same cancer type, an overall expression heatmap was generated from the average D value for addressing the significance of the miRNA in multiple cancers. In addition, hyperlinks to the computationally predicted targets for each miRNA by miRanda [33], PicTar [34] and TargetScan [35] were also provided.

This release of dbDEMC contains 607 differentially expressed miRNAs (590 mature miRNAs and 17 precursor miRNAs) from 14 types of cancer, including breast cancer, colon cancer, esophageal carcinoma, head and neck squamous cell carcinoma, hepatocellular carcinoma, lung cancer, lymphoma, medulloblastoma, prostate cancer, renal carcinoma, uterus carcinoma, glioblastoma, multiple myeloma and meningioma. The expression information of the 132 miRNAs retrieved from low-throughput experiments was also included.

### Database architecture

The expression information of miRNAs was imported into the database powered by MySQL. The web interface was implemented using PHP language, and Apache was used as the HTTP server.

### Web interface

**Figure 1:**
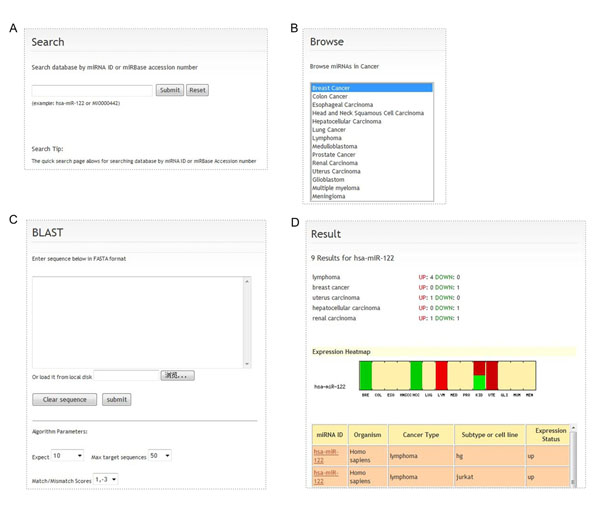
**The web interface of dbDEMC.** (A) Key word search. miRNAs can be searched via miRNA ID or miRBase accession number. (B) Browse database, users can browse the miRNAs of specific cancers. (C)  BLAST. An easy-to-use interface was created for parameter selection. (D) A part of search result page, consisting of a summary, an expression heatmap and the expression status.

dbDEMC provides different ways to navigate the database. For text search, dbDEMC supports miRNA searching via miRNA IDs or miRBase accession numbers (Figure 1A). Users can also select a specific cancer type to browse differentially expressed miRNAs (Figure 1B). A customized BLAST tool is also available for sequence similarity search [36]. It is useful to determine whether the query sequence is overlapped with the existing miRNAs in the database (Figure 1C). The results page for search and browse lists the basic information on matching, such as the miRNA ID, summary, cancer type, related cancer subtypes or cell lines, and expression statues (up- or down-regulation) (Figure 1D).

**Figure 2:**
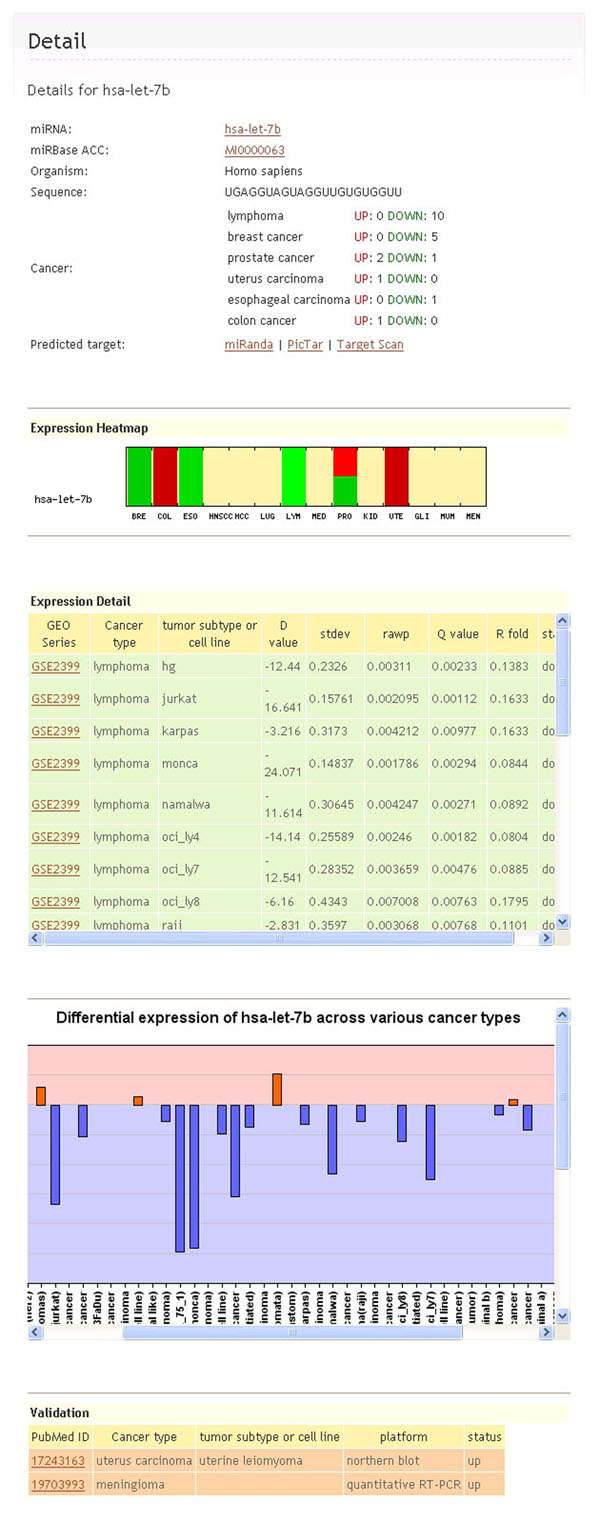
**Detailed page of an example miRNA record.** The whole page consists of five major parts: a summary, an expression heatmap, statistical details, the D value across signatures, and the low-throughput data for validation.

Detailed expression information of a specific miRNA can be accessed via the hyperlink of miRNA ID (Figure 2). Besides a summary about miRBase accession number, miRNA sequence and expression change in different cancers is also presented. A heatmap was generated to visualize such changes. Results of Differential expression ratio (D value), stdev, Q value and R fold from different expression profiles of the miRNA were displayed so that the degree of deregulated information can be evaluated. In addition, the miRNA expression information retrieved from low-throughput experiments were presented if it was available.

## Conclusion

As a novel class of molecular biomarker, cancer-related miRNAs is of great importance for clinicians and cancer immunologists to detect. To achieve this goal, we developed dbDEMC, a database that allows for an effective retrieval of microRNA expression information in different cancers. In summary, we believe this database will greatly facilitate the identification of cancer-related miRNAs and the discrimination and determination of different cancer types, as well as their lineage during development. The dbDEMC may be accessed through http://159.226.118.44/dbDEMC/index.html. All the information is freely available to users. As the number of miRNAs and their corresponding expression profiles from different cancers increase, this database will be continually updated.

## Discussion

**Figure 3:**
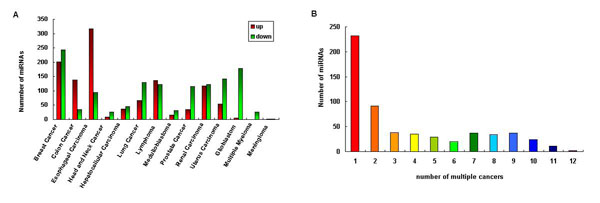
**Data content of dbDEMC.** (A) The number of the differentially expressed miRNAs in each cancer type. (B) The number of the differentially expressed miRNAs in multiple cancers.

The dbDEMC includes miRNAs that have the expression information in different cancers determined by statistical analysis of microarray data. Until now, a total of 607 miRNAs were identified as deregulated in one or more cancer types, comprising more than half of the miRNAs discovered in humans [32]. Figure 3A illustrates the number of differentially expressed miRNAs for each cancer type. Taking breast cancer as an example, a total of 371 miRNAs were identified to be differentially expressed, where 202 miRNAs were up-regulated and 243 miRNAs were down-regulated. Analysis results from different data sources indicated that the expression levels of a specific miRNA are often different and even contradict with each other among many experiments. The different cancer subtypes, cell lines and the experiment platforms used may be the explanation for these differences. In this sense, the integration of the data from different source provides more rich and reliable miRNA expression information in cancers.

We also compared the content of dbDEMC with cancer related miRNAs in miR2Disease. Keywords including the "cancer", "carcinoma", "lymphoma" and "leukemia" were used to search cancer related miRNAs in miR2Disease and a total of 303 mature miRNAs were retrieved. By comparison of the 590 mature miRNAs included in dbDEMC, 253 (42.9%) shared with the above result, which suggests that dbDEMC will be an important complement to other existing databases.

As candidate biomarkers for diagnosis, treatment and prognosis of human cancers, the differentially expressed miRNAs in dbDEMC can be used to infer miRNA-cancer relationships. Figure 3B demonstrates the number of the deregulated miRNAs in multiple cancers. 232 miRNAs (39.3%) have differential expression in only one cancer, while 267 miRNAs (45.3%) have differential expression in no less than three cancers. The most prevalent miRNAs are miR-143 and miR-214, both of which are associated with twelve cancers types. miR-143 is down-regulated in ten cancers and has conflicting results in two cancers, whereas miR-214 is down-regulated in eight cancers, up-regulated or has conflicting expression results in two cancers. Common miRNAs shared by multiple cancers with similar deregulation status may suggest they have common regulatory mechanisms and can be regarded as therapeutic targets. To provide more accurate and reliable miRNA expression information in multiple cancers, a meta-profìling analysis of the expression results in the 48 microarray data sets was performed following Daniel *et al *[37]. The meta-profìling analysis addresses significant intersections of miRNAs shared by different expression signatures, which are defined here as the statistical results of microarray experiments. For a set of S differential expression signatures, miRNAs were first sorted by the number of signatures that they presented. Then the number of miRNAs associated with each possible number of multiple signatures were calculated (*N_0_, N_1_, N_2_...N_S_*). Subsequently, random permutations were performed in which the D values were randomly assigned to each miRNA within each signature, so that an index of the number of the miRNAs associated with the number of random signatures (*E_0_, E_1_, E_2_...E_S_*) was generated. A minimum meta-false discovery rate (mFDR_min_) was determined to assess the significance of the interaction among signatures:

mFDR_min_ = MINIMUM ([*E_i_* + 1]/[*N_i_*]) (for i = 0 ... S)

**Figure 4:**
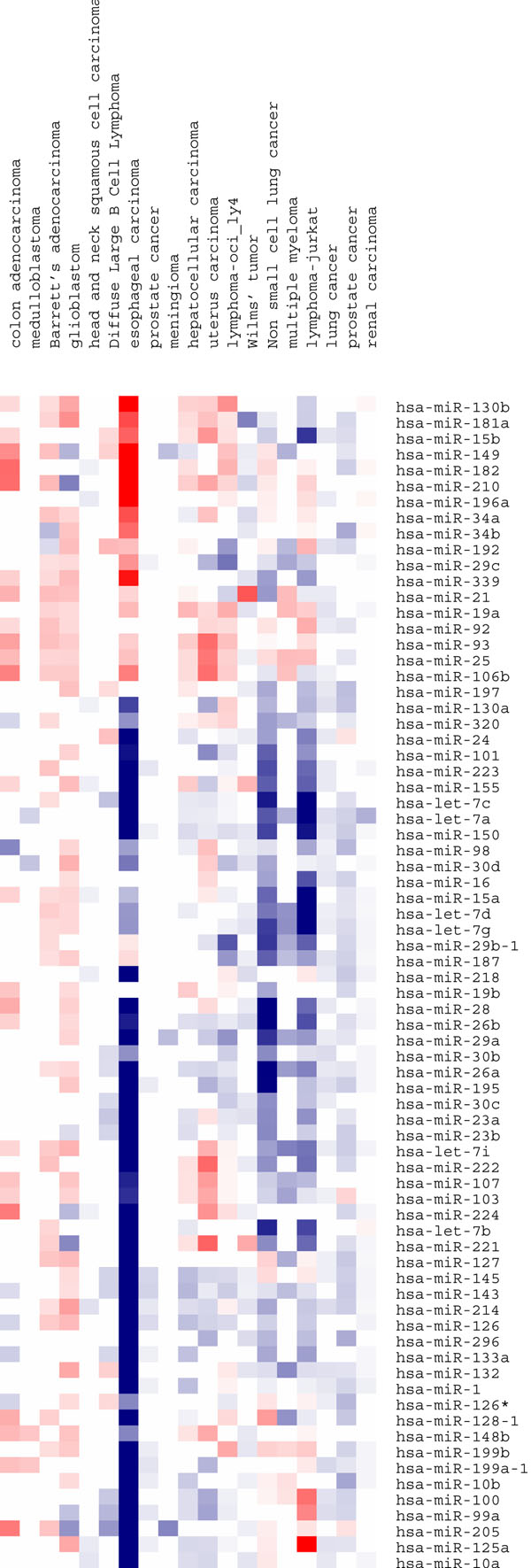
**Meta-signature of multiple miRNAs.** Seventy-four miRNAs have different expression level in at least 20 "cancer vs normal" signatures. Twenty cancer types were selected for this figure. Red boxes signify significant up-regulation in cancers compared to normal tissues, blue boxes signify significant down-regulation in cancers compared to normal tissues, and white boxes signify no significance or missing data.

miRNAs were defined as significantly differently among multiple signatures if mFDR_min_ < 0.1. The above steps were repeated as the number of miRNAs was iteratively lowered by 50% within each signature according to the D value until the mFDR_min_ threshold was reached. At the initial significant threshold, a total of 107 miRNAs were deregulated in more than 15 signatures, 74 miRNAs were deregulated in more than 20 signatures and one miRNA was deregulated in 46 signatures, whereas for the random simulation, no miRNA was found to be deregulated in more than 15 signatures. This indicates the statistically significant multi-signature expression of the 107 miRNAs (mFDR_min_ = 0.034) in multiple cancers. The meta-signature for the 74 miRNAs was depicted in Figure 4. Many of these miRNAs have been previously demonstrated to be associated with cancers. For example, miR-15 and miR-16 were shown to be down-regulated in B-cell leukemia and involved in the development of multi-drug resistance in gastric cancer [15,38]. miR-143 and miR-145 are down-regulated in colon cancer and B-cell malignancies [39,40]. A high expression level of miR-196a may induce colorectal cancer and Barrett's esophagus [41,42]. Moreover, many miRNAs were shown to be associated with the development of breast cancer or its drug resistance, including miR-125a, miR-96, miR-182, miR-221 and miR-222 [43-45]. The common expression profiling of miRNAs suggests that there a common regulatory mechanism for cell division and proliferation may exits. Alternation of transcriptional features may contribute to unregulated cell division and neoplastic transformation of the cancer cell. On the other hand, these small numbers of miRNAs may represent important therapeutic targets against cancer.

## Competing interests

The authors declare that they have no competing interests.

## Authors' contributions

Haitao Zhao, Yi Zhao and Yang Zhong conceived the concept of dbDEMC. Changning Liu and Shunmin He collected and compiled data from literature and public databases. Gang Sun and Qian Gao performed the analysis. Zhen Yang and Fei Ren constructed the database and compiled the draft of the manuscript. Lei Yao incorporated the results and participated in the design of the database. Yangde Zhan, Ruoyu Miao and Ying Cao revised the manuscript. All authors read and approved the final manuscript.
